# Deficiency of TPPP2, a factor linked to oligoasthenozoospermia, causes subfertility in male mice

**DOI:** 10.1111/jcmm.14149

**Published:** 2019-01-24

**Authors:** Feng Zhu, Peipei Yan, Jingjing Zhang, Yiqiang Cui, Meimei Zheng, Yiwei Cheng, Yueshuai Guo, Xiaoyu Yang, Xuejiang Guo, Hui Zhu

**Affiliations:** ^1^ State Key Laboratory of Reproductive Medicine, Department of Histology and Embryology Nanjing Medical University Nanjing China; ^2^ Department of Pathology Women’s Hospital of Nanjing Medical University, Nanjing Maternity and Child Health Care Hospital Nanjing China; ^3^ Clinical Center of Reproductive Medicine The First Affiliated Hospital of Nanjing Medical University Nanjing China

**Keywords:** energy metabolism, male subfertility, oligoasthenozoospermia, sperm function, TPPP2

## Abstract

Oligoasthenozoospermia is a major cause of male infertility; however, its etiology and pathogenesis are unclear and may be associated with specific gene abnormalities. This study focused on *Tppp2* (tubulin polymerization promoting protein family member 2), whose encoded protein localizes in elongating spermatids at stages IV‐VIII of the seminiferous epithelial cycle in testis and in mature sperm in the epididymis. In human and mouse sperm, in vitro inhibition of TPPP2 caused significantly decreased motility and ATP content. Studies on *Tppp2* knockout (KO) mice demonstrated that deletion of TPPP2 resulted in male subfertility with a significantly decreased sperm count and motility. In *Tppp2* ^−/−^ mice, increased irregular mitochondria lacking lamellar cristae, abnormal expression of electron transfer chain molecules, lower ATP levels, decreased mitochondrial membrane potential and increased apoptotic index were observed in sperm, which could be the potential causes for its oligoasthenozoospermia phenotype. Moreover, we identified a potential TPPP2‐interactive protein, eEf1b (eukaryotic translation elongation factor 1 beta), which plays an important role in protein translation extension. Thus, TPPP2 is probably a potential pathogenic factor in oligoasthenozoospermia. Deficiency of TPPP2 might affect the translation of specific proteins, altering the structure and function of sperm mitochondria, and resulting in decreased sperm count, motility and fertility.

## 1 INTRODUCTION

The World Health Organization estimates that 50 million couples worldwide are confronted with infertility.^1^ Infertility now is a global problem afflicting approximately 15% of the couples of reproductive age and in 50% of cases, the attributing factor is linked to men.^2^, ^3^ Among these infertile men, 18% specifically exhibit asthenozoospermia and 63% exhibit asthenozoospermia associated with oligo‐ and/or terato‐zoospermia.^4^

Sperm is the ultimate executor in male reproduction. Sperm counts and motility are the most important indicators of male fertility and only a certain amount of normal progressively motile sperm can ensure the fertilization of an egg. Oligoasthenozoospermia now is a major factor in male infertility.

However, the causes of oligoasthenozoospermia are complex.^5^, ^6^, ^7^ Until now, the etiology and pathogenesis of the disease have been unclear, thus treatment has many limitations and shows low efficacy.^7^, ^8^, ^9^ In recent years, although assisted reproductive technology has effectively improved the pregnancy rate of patients with oligoasthenozoospermia, it has not fundamentally improved the quality of patients' sperm and increases the risk of foetal growth restriction, low birth weight and neonatal diseases.^10^ Multilayered synergetic clinical and basic research should therefore be performed to reveal the etiology and pathogenesis underlying oligoasthenozoospermia, representing the majority of infertility cases.

Oligoasthenozoospermia is a manifestation of spermatogenesis and functional maturation disorders. Spermatogenesis is a complex process in the testis including several important biological events, such as the proliferation of spermatogonia, the meiosis of spermatocytes and spermiogenesis. The spermatozoa produced in the testes are not yet functionally mature. They have to transit through the epididymis and then acquire their forward motility and their ability to efficiently encounter the egg and its layers.^11^ On this basis, revealing the regulatory mechanism of spermatogenesis/sperm function would help to clarify the pathogenesis of oligoasthenozoospermia and is an important issue in male reproductive research.

In recent years, proteomic studies of the human organs have increased our knowledge and understanding of proteins’ function. Proteomics is the systematic study of the proteins contained in a specific cell, tissue or organ.^12^, ^13^, ^14^ In the process of spermatogenesis and functional maturation, the orderly and specific expression of a gene/protein network plays an important regulatory role and a series of genes/proteins have been identified as related to spermatogenesis and/or sperm function such as T‐complex‐associated‐testis‐expressed 1,^2^ MORC family CW‐type zinc finger 2B,^15^ ER membrane protein complex subunit 10,^16^ B lymphoma Mo‐MLV insertion region 1 homolog (mouse),^17^ and spermatid‐specific protein 411.^18^ It is necessary to identify more genes or proteins involved in these processes. In our previous work, a human testis and sperm proteome database was constructed using a proteomics research platform, providing an overall view of the testis and sperm proteins, which helped us to gain insights into the multi‐level, multi‐perspectives of the protein networks active during spermatogenesis/sperm function.^19^, ^20^ In the present study, we concentrated on a protein expressed in human testis and sperm named tubulin polymerization‐promoting protein family member 2 (TPPP2), which has a homologous gene in mice and thus it was feasible to explore the regulatory mechanism of spermatogenesis/sperm function in detail using a *Tppp2* knockout mouse model.

There are three TPPP paralogs in the human genome denoted as TPPP/p25, TPPP2/p18 and TPPP3/p20 (shortened to TPPP1, TPPP2 and TPPP3, respectively), indicating their molecular mass.^21^, ^22^, ^23^ TPPP1 and TPPP3 have been the subjects of several studies. TPPP3 shares the microtubule associated protein‐like features of TPPP1, binding and polymerizing tubulin and stabilizing microtubules.^24^, ^25^, ^26^ TPPP1 is a brain‐specific protein that is involved in brain developmental processes. Under pathological conditions, TPPP1 is enriched in glial and neuronal inclusions in the synucleinopathies of Parkinson's disease and multiple system atrophy.^26^, ^27^, ^28^ TPPP3 is involved in developmental processes of the musculoskeletal system and is a specific marker of the differentiating tendon sheath and synovial joints.^29^ These results suggested that TPPP family members might have significant tissue specific expression and thus play an important role in the development and function of specific tissues. Interestingly, it seems that TPPP2 is different from TPPP1 and TPPP3, which are linked to microtubules. Although there has been no study on the function of TPPP2 to date, preliminary experiments in vitro showed that TPPP2 was distributed homogeneously within the cytosol of transfected HeLa cells.^24^ In the present study, we found TPPP2 in our human testis and sperm proteome database, which indicated that TPPP2 is likely to play an important role in spermatogenesis and/or sperm function.

This study aimed to use human samples and KO mouse models to explore the function and mechanism of TPPP2. Our results will provide a new perspective on the complex spermatogenesis regulatory networks. We also anticipate providing new targets for male infertility diagnosis and for the development of male contraceptives.

## 2 MATERIALS AND METHODS

### 2.1 Animals

*Tppp2* KO mice were generated via Cas9/RNA‐mediated gene targeting as described previously.^30^ All mice were housed in a specific pathogen‐free animal facility with a light:dark cycle of 12:12. All the animal experiments in this study were approved by the Institutional Animal Care and Use Committees of Nanjing Medical University, Nanjing, China.

### 2.2 Detection of sperm function parameters

#### 2.2.1 Assessment of sperm motility and sperm count

Sperm were extracted and incubated in human tubal fluid (HTF) medium (EasyCheck, Nanjing, China) at 37°C. Sperm samples were diluted and a 10‐μl aliquot of the sperm sample was evenly distributed on a glass chamber slide and analysed using a Computer Assisted Sperm Analyzer (CASA) via the IVOS II™ system (Hamilton Thorne, Beverly, MA, USA).

#### 2.2.2 Assessment of capacitation and acrosome reaction

The human sperm prepared as described above were capacitated for 5 hours in TYH medium (EasyCheck, Nanjing, China) at 37°C and 5% CO_2_. Calcium ionophore A23187 (final concentration 10 µmol/L; Sigma‐Aldrich, St. Louis, MO, USA) was then added to the capacitated sperm for 30 minutes to induce the acrosome reaction. The percentage of capacitated and acrosome reacted human sperm was evaluated by staining with chlortetracycline (Sigma‐Aldrich). At least 200 sperm were counted under an LSM700 confocal microscope (Carl Zeiss AG, Gottingen, Germany).

#### 2.2.3 In vitro fertilization assay

The cumulus‐oocyte complexes (COCs) were isolated from superovulated female mice 13 hours after Human chorionic gonadotropin (HCG, Sansheng Biological Technology, Ningbo, China) injection. The COCs in HTF medium (EasyCheck) were mixed with capacitated epididymal sperm and incubated at 37°C under 5% CO_2_, then placed in KSOM medium (EasyCheck) and undisturbed cultivation was performed for 1 day. Developing embryos were determined microscopically based on progression to the two‐cell stage.

#### 2.2.4 Measurement of sperm ATP

Sperm samples were washed twice, resuspended in lysis buffer, vortexed and then placed on ice. ATP was measured using luminometric methods with commercially available luciferin/luciferase reagents according to the manufacturer's instructions (ATP Assay Kit, Beyotime Biotechnology, Shanghai, China). An average of 3 × 10^7^ sperm were used for ATP analysis.

#### 2.2.5 MMP assay

The mitochondrial membrane potential (MMP) was assessed using a JC‐1 Mitochondrial Membrane Potential Detection Kit (Beyotime Biotechnology). Briefly, sperm were incubated with an equal volume of JC‐1 staining solution at 37°C for 20 minutes and rinsed twice with phosphate‐buffered saline. Sperm treated with 10 µmol/L carbonyl cyanide 3‐chlorophenylhydrazone, which is a protonophore that can cause dissipation of MMP, were used as a positive control. MMPs, were monitored by determining the relative amounts of dual emissions from mitochondrial JC‐1 monomers or aggregates using an LSM700 confocal microscope (Carl Zeiss AG) and flow cytometer. Mitochondrial depolarization was indicated by an increase in the green/red fluorescence intensity ratio.

### 2.3 Assessment of fertility

To determine the fertility of the animals, a male was mated with two females. The female mouse was separated from the male after the formation of a normal vaginal plug was detected. The number of offspring produced per female was recorded for 6 months.

### 2.4 Histological analysis

The testes and epididymis tissues were dissected, fixed in modified Davidson's Fluid and embedded in paraffin. Sections were cut and then stained using hematoxylin and eosin and Periodic Acid‐Schiff's technique following our previously published protocols.^2^

The seminiferous epithelium cycle (SEC) was characterized using morphological criteria into various developmental stages, primarily based on the form and shape of the acrosome and to a lesser extent, on spermatid head shape and the degree of chromatin condensation. Each stage with a distinct ordering of cell associations along the length of the seminiferous tubule was designated by Roman numerals.^31^ All spermatocytes and round spermatids cell nucleoli were counted per animal in five round seminiferous tubule cross‐sections at stages VII‐VIII of the cycle.

For ultrastructural examination, 2.5% glutaraldehyde‐fixed sperm were post‐fixed with 2% (wt/vol) OsO4 and embedded in Araldite. Ultrathin sections were stained with uranyl acetate and lead citrate and analysed using electron microscopy (JEOL, Tokyo, Japan).

### 2.5 Quantitative real‐time PCR analysis

Total RNA was extracted from mouse tissues or cells using Trizol (Thermo Fisher Scientific, Waltham, MA, USA) following the manufacturer's instructions. cDNA synthesis was carried out using a Prime Script™ RT Master kit (Takara Bio, Otsu, Shiga, Japan). The SYBR Premix Ex Taq II kit (Takara Bio) was used for real‐time polymerase chain reaction. Reactions were performed according to the manufacturer's protocol. Primer sequences are listed in Supplemental Table S1.

### 2.6 Western Blotting analysis

Protein levels in mouse tissues or cells were determined by western blotting using the following antibodies: anti‐TPPP2 (Abcam, 121215; 1:1000), anti‐green fluorescent protein (GFP) (EnoGene, E12‐026‐4; 1:3000), anti‐β‐ACTIN (Merck Millipore, MAB1501; 1:7500), anti‐Caspase3 (Proteintech, 19677‐1‐AP; 1:500), anti‐Bax (Proteintech, 50599‐2‐Ig; 1:500), anti‐Bcl‐2 (Proteintech, 12789‐1‐AP; 1:500) and anti‐Eef1b (Proteintech, 10483‐1‐P; 1:1000).

### 2.7 Immunohistochemistry and immunofluorescence analysis

Prepared mouse tissues or cells were blocked in 1% bovine serum albumin and then incubated overnight at 4°C with anti‐TPPP2 antibodies (Abcam, 121215; 1:250). The samples were then incubated with secondary antibodies at room temperature for 2 hours, following our previously published protocols.^17^ Slides were viewed under a bright‐field microscope (ZEISS Axio Skop plus2) for immunohistochemical analysis and under a LSM700 confocal microscope (Carl Zeiss AG) for immunofluorescent analysis.

### 2.8 Immunoprecipitation from cell culture extracts

HEK293T cells were transfected with GFP‐tagged TPPP2 fusion plasmids using Lipofectomine 2000 (Thermo Fisher Scientific). Two days after transfection, cells were lysed using Pierce IP Lysis Buffer (Thermo Fisher Scientific) supplemented with 1% (vol/vol) protease inhibitor mixture (Selleck, Houston, TX, USA) for 40 minutes at 4°C and then clarified using centrifugation at 12 000 *g* for 20 minutes. The lysates were precleared with 20 μL of Protein A/G magnetic beads (Selleck, Houston, TX, USA) for 1 hour at 4°C. The precleared lysates were incubated overnight with anti‐GFP antibodies at 4°C. Lysates were then incubated with 50 μL of Protein A/G magnetic beads for 3 hours at 4°C. The beads were washed three times with IP Lysis Buffer and eluted with Pierce IgG Elution Buffer (Thermo Fisher Scientific). Samples were boiled in SDS loading buffer before being subjected to SDS/PAGE.

### 2.9 ***Mass spectrometry*** ***analysis***

For mass spectrometry analysis, elution was followed by in‐solution digestion. Briefly, elution was first achieved using ultrafiltration with a 10‐kDa filter membrane in protein extraction buffer [8 mol/L urea, 75 mmol/L NaCl, 50 mmol/L Tris, pH 8.2, 1% (vol/vol) EDTA‐free protease inhibitor, 1 mmol/L NaF, 1 mmol/L β‐glycerophosphate, 1 mmol/L sodium orthovanadate, 10 mmol/L sodium pyrophosphate]. Cysteine residues were reduced with dithiothreitol (DTT) at a 5 mmol/L final concentration for 25 minutes at 56°C followed by alkylation in 14 mmol/L iodoacetamide for 30 minutes at room temperature in the dark. Unreacted iodoacetamide was quenched with DTT for 15 minutes. Lysates were then diluted down to 1.6 mol/L urea with 25 mmol/L Tris, pH 8.2. CaCl_2_ was added to a final concentration of 1 mmol/L and digested overnight at 37°C with trypsin at a concentration of 5 ng/µL. Trifluoroacetic acid at a final concentration of 0.4% was added to stop the digestion. The peptides were desalted using OASIS HLB Extraction Cartridges (Waters, Milford, MA, USA) before mass spectrometric analysis. We performed MS analyses according to previously published experimental procedures.^2^ All raw files were searched using MaxQuant software (version 1.3.0.5, Martinsried, Germany) against the UniProt human proteome database.

### 2.10 Statistical analysis

All assays were repeated at least three times. Statistical analyses were evaluated using SPSS19.0. The statistical significance of differences in mean values was assessed using a *t* test. Values of *P* < 0.05 were considered statistically significant.

## 3 RESULTS

### 3.1 Histological expression and cellular localization of TPPP2

**Figure 1:**
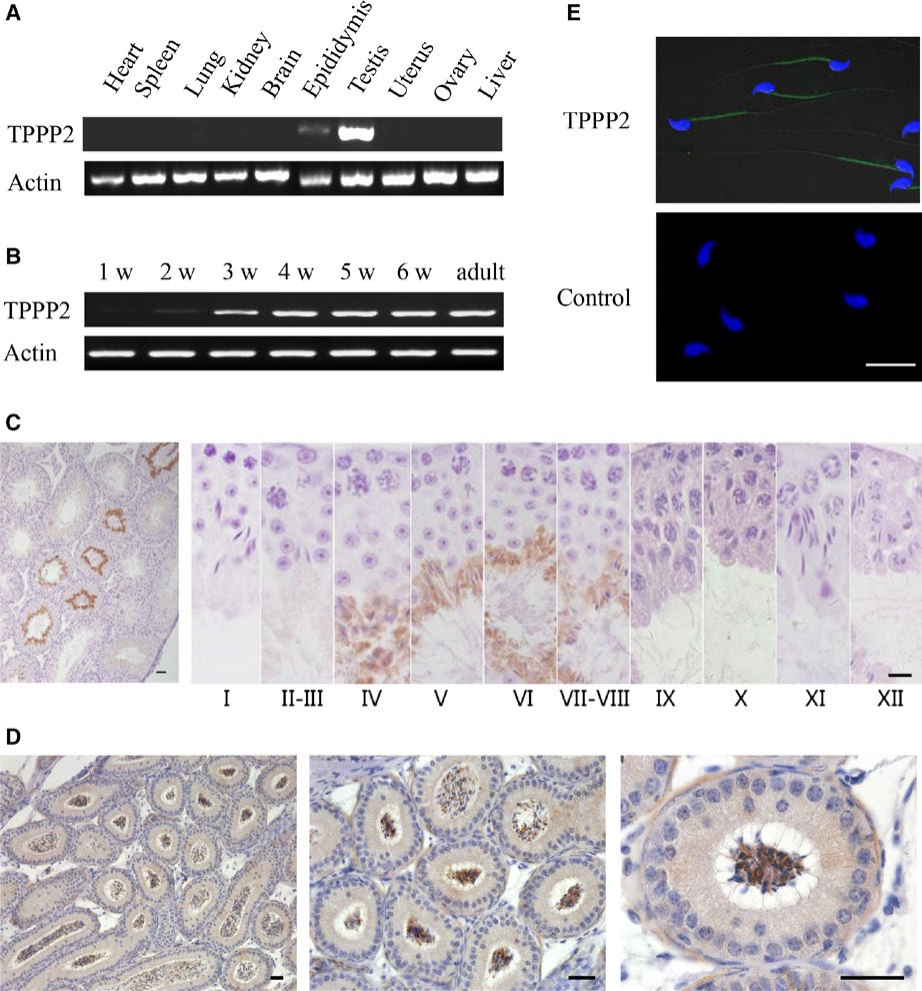
Distribution of TPPP2 in mouse tissues. (A) The presence of *Tppp2* mRNA was evaluated in various tissues from adult mice using reverse transcription polymerase chain reaction (RT‐PCR). *Tppp2* mRNA was detected in the testis and epidydimis. β‐Actin was amplified as an internal control. (B) The testicular *Tppp2* mRNA expression profile was tested at the indicated time points after birth using RT‐PCR. β‐Actin was amplified as an internal control. *Tppp2* mRNA expression was markedly increased at the third week. (C) Immunohistochemical localization of the TPPP2 protein in adult mouse testis. Each image shows a stage of the seminiferous epithelial cycle denoted by Roman numerals at the bottom of each image. (D) Immunohistochemical localization of the TPPP2 protein in adult mouse epididymis. (E) Mature sperm were subjected to immunofluorescent microscopy using an anti‐TPPP2 antibody (green) and Hoechst staining (blue), as described in the Materials and Methods. TPPP2 was expressed in the middle piece of the mature sperm. Scale bars, 20 µm

RT‐PCR revealed the distribution of *Tppp2* mRNA in various tissues of mice including the heart, spleen, lung, kidney, brain, epidydimis, testis, uterus, ovary and liver. As shown in Figure 1A, *Tppp2* was only expressed in male reproductive organs including the testis and epidydimis. Testis maturation is age‐dependent and the first wave of spermatogenesis takes place within 35 post‐natal days in mice. We examined the *Tppp2* mRNA abundance in testis of mice at 1, 2, 3, 4, 5 and 6 post‐natal weeks and in adults. The results showed that *Tppp2* mRNA sharply increased in the third week post‐natal (Figure 1B), when the spermatids have formed.

Further immunohistochemical analysis of adult mouse testis revealed that TPPP2 was restrictively expressed at stages IV‐VIII of the seminiferous epithelial cycle corresponding to the spermatids at steps 15‐16 of spermiogenesis (Figure 1C), when the morphology of elongated spermatids has basically formed. Such a specific expression pattern might indicate an important physiological role of TPPP2 during sperm function in mice.

Immunohistochemical analysis of adult mouse epididymides showed that TPPP2 was expressed in sperm, rather than in epithelial cells (Figure 1D). Then, immunofluorescence analysis was carried out to identify the localization of TPPP2 in mature sperm. The results showed that TPPP2 was expressed in the middle piece of the sperm tail (Figure 1E).

### 3.2 Inhibition of TPPP2 impaired sperm motility

**Figure 2:**
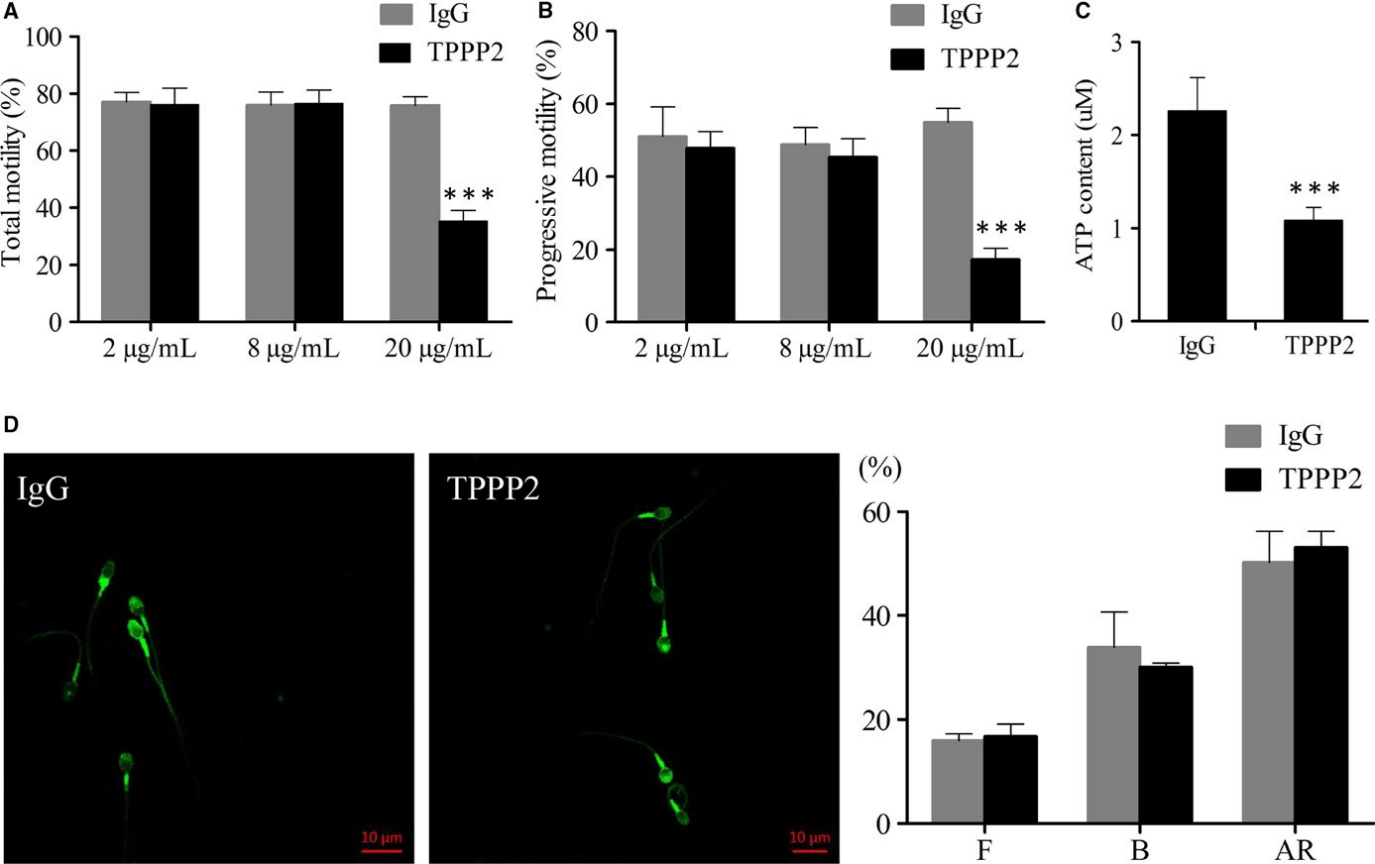
Changes in human sperm treated with anti‐TPPP2 antibodies. Sperm concentration = 30 × 10^6^ sperm/mL. (A‐B) 2, 8 and 20 µg/mL of TPPP2 antibody were respectively co‐incubated with sperm for 30 min and the corresponding concentrations of IgG were co‐incubated with sperm as controls. Motility was evaluated by counting 200 spermatozoa in randomly selected fields. Data are presented as the mean ± SD (n = 10). ***P < 0.001. (C) ATP contents were further measured in sperm between the IgG and 20 µg/mL TPPP2 groups (n = 10). ***P < 0.001. (D) Assessment of capacitation and acrosome reaction. Capacitated sperm underwent A23187‐induced acrosome reaction. Data are presented as the mean ± SD (n = 3). F: uncapacitated sperm; B: capacitated sperm; AR: acrosome reacted sperm

To determine whether TPPP2 is associated with sperm function, human sperm were co‐incubated with anti‐TPPP2 antibodies at different concentrations (2, 8 and 20 µg/mL) for 30 minutes and the corresponding concentrations of IgG were co‐incubated with sperm as controls. The results showed that sperm motility in the 20 µg/mL anti‐TPPP2 antibody group was reduced significantly compared with that in the control group (*P < *0.001); the sperm motility of the 2 µg/mL and 8 µg/mL anti‐TPPP2 antibody groups showed no significant differences (Figure 2A‐B).

We examined the sperm ATP content of the 20 µg/mL anti‐TPPP2 antibody treatment group with significantly decreased sperm motility. The results showed that the sperm ATP content in the antibody treatment group was significantly lower than that in the control group (Figure 2C) (*P < *0.001), suggesting that the decrease in sperm motility after incubation might be caused by a decrease in the ATP content.

In addition, human sperm was incubated with IgG antibody (20 µg/mL) and TPPP2 antibody (20 µg/mL) and then assayed by CTC staining to determine the percentage of capacitated and acrosome‐reacted sperm. The results showed that the percentages of capacitated and acrosome‐reacted sperm between these two groups were not significantly different (Figure 2D).

**Figure 3:**
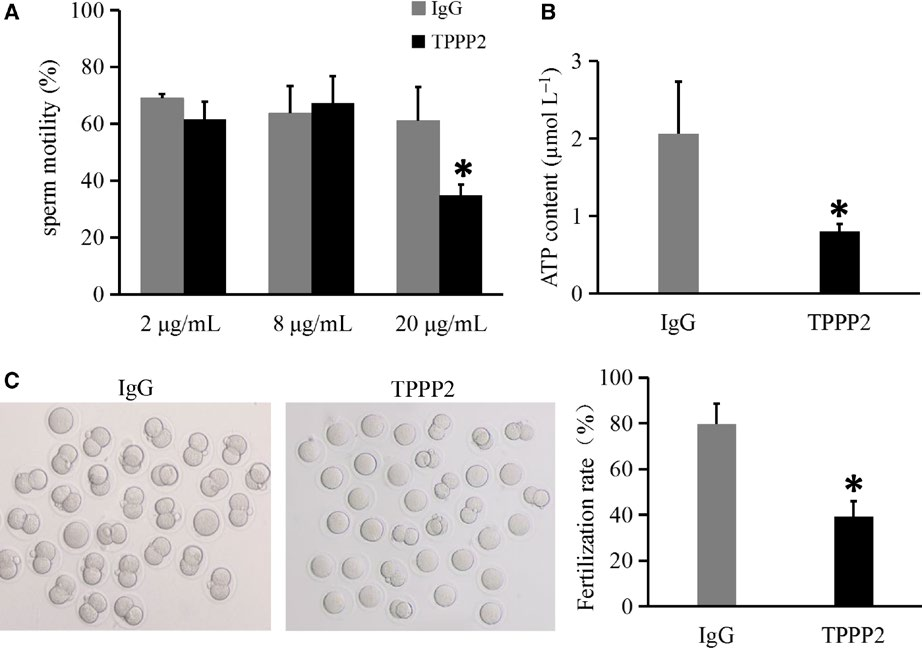
Changes in mouse sperm treated with anti‐TPPP2 antibodies. (A) 2, 8 and 20 µg/mL of TPPP2 antibody respectively, were co‐incubated with sperm for 30 min and the corresponding concentrations of IgG were co‐incubated with sperm as controls. Sperm samples were analysed using a Computer Assisted Sperm Analyzer (CASA) (n = 3), *P < 0.05. (B) ATP contents were further measured in sperm between the IgG and 20 µg/mL TPPP2 groups (n = 3), *P < 0.05. (C) When cumulus‐intact wild‐type (WT) eggs were inseminated with sperm from the IgG and 20 µg/mL TPPP2 group, the two‐cell cleavage rate at 24 h in 20 µg/mL TPPP2 group was significantly lower than that in the IgG group (n = 3), *P < 0.05

The effects of anti‐TPPP2 antibody on mouse sperm were also evaluated. As shown in the images, the 20 µg/mL anti‐TPPP2 antibody caused significantly reduced sperm motility with a significantly decreased ATP content (Figure 3A‐B) (*P < *0.05). Moreover, this adverse effect appeared to further affect the in vitro fertilization rate. The fertilization rate in the 20 µg/mL anti‐TPPP2 antibody treatment group was significantly lower than that in the IgG treatment group (Figure 3C) (*P < *0.05).

### 3.3 Generation of TPPP2 gene KO mice

**Figure 4:**
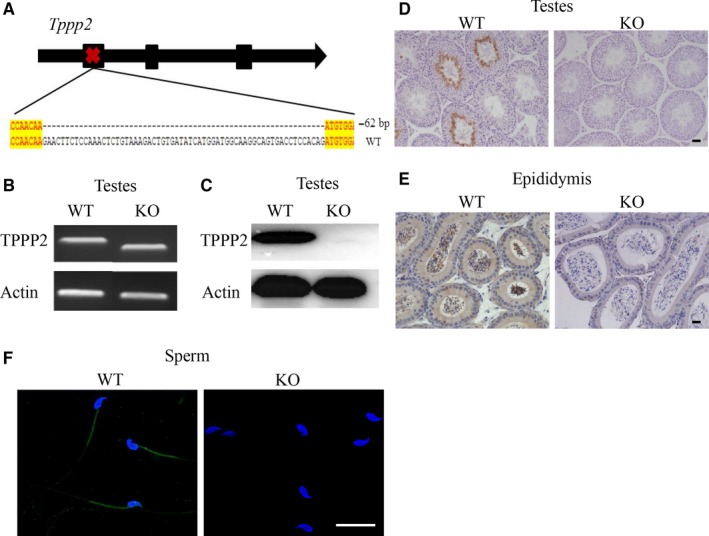
Generation of *Tppp2* gene knockout (KO) mice. (A) Schematic strategies for the generation of KO mice using CRISPR/Cas9 technology. 62 bp of *Tppp2* were deleted from Exon 1 (n = 3). (B) Genotype of each KO mouse was confirmed using PCR (n = 3). (C) TPPP2 levels in the testes of KO and wild‐type (WT) mice were evaluated using western blotting. β‐actin was used as a loading control (n = 4). (D‐F) TPPP2 levels in the testes and sperm of KO and WT mice were evaluated using immunohistochemical and immunofluorescent analysis. Scale bars, 20 µm (n = 3)

Gene editing using CRISPR/Cas9 technology combined with microinjection technology was used to construct *Tppp2* gene KO mice. The representative Sanger sequence image for the verification of the KO mouse is shown in Figure 4A. After the extraction of DNA, PCR amplification was performed with specific primers (Figure 4B). The results confirmed the deletions of targeted regions in *Tppp2* ^−/−^ male mice, leading to a frame‐shift mutation of *Tppp2*.

**Figure 5:**
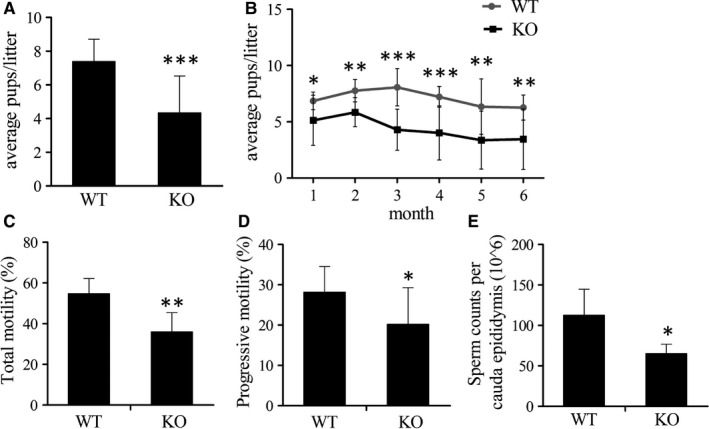
Fertility assay and sperm parameters. (A‐B) Each male was bred with two females. WT, wild‐type. Knockout (KO), *Tppp2* ^−/−^ male mice. Tests were performed for average pups/litter between the WT and KO groups. Data are presented as mean ± SD (for the WT group, n = 9; for the KO group, n = 10). *P < 0.05, **P < 0.01, ***P < 0.001. (C‐D) Percentage of motile and progressively motile sperm from WT and KO males (n = 6). (E) Sperm counts per cauda epididymis of WT and KO males (n = 6)

**Figure 6:**
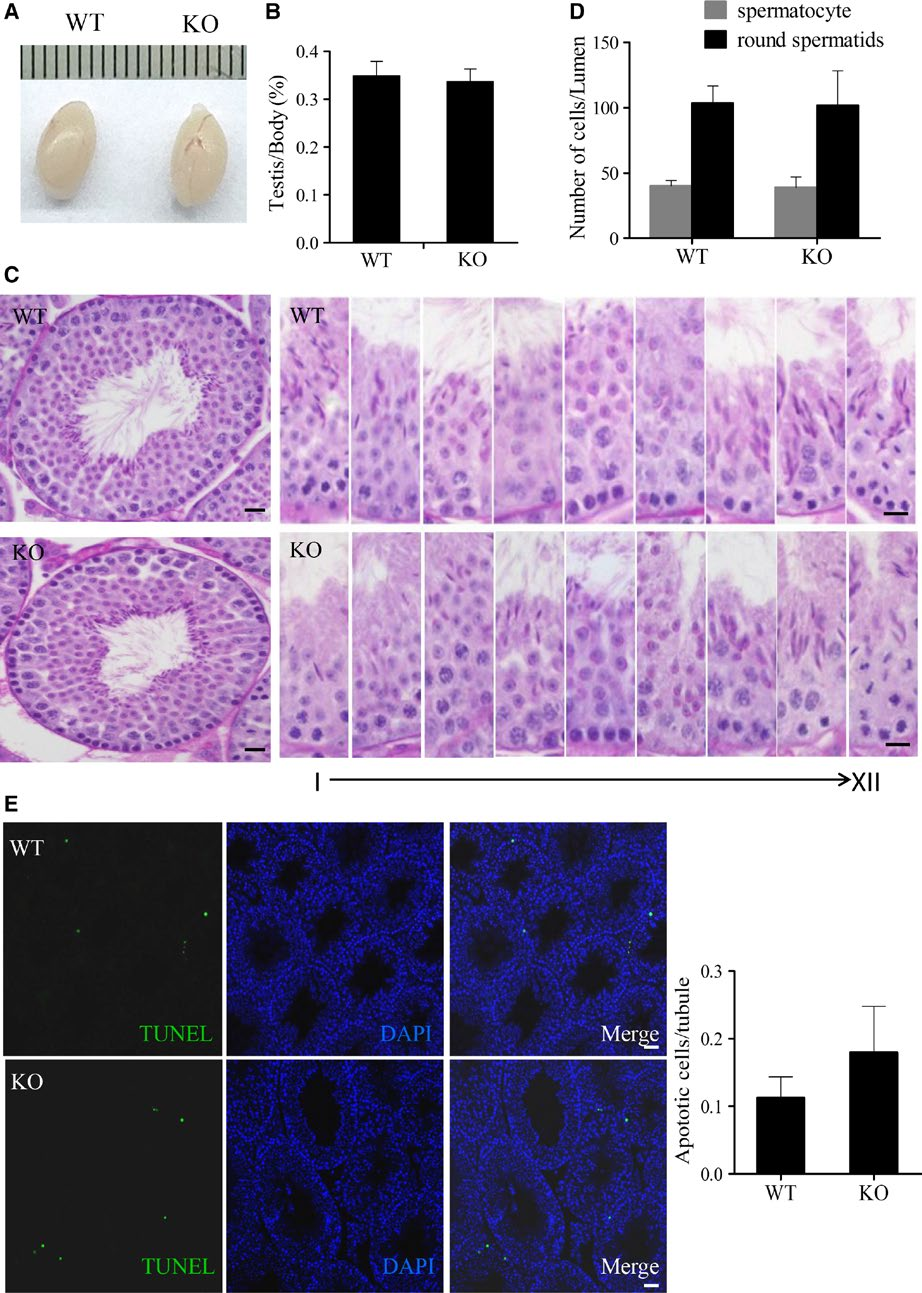
Spermatogenesis in wild‐type (WT) and knockout (KO) mice. (A) Morphology and size of WT and KO mice testes. (B) Testis/body weight ratio for WT and KO mice (n = 6). (C) Periodic Acid‐Schiff's‐stained sections of testes from WT and KO male mice. Each image exhibits a stage of the seminiferous epithelial cycle denoted by Roman numerals at the bottom of the image. All showed normal spermatogenesis. (D) The number of spermatocytes and round spermatids from the testes of WT and KO males were counted as shown (n = 5). Scale bars, 20 µm. (E) Terminal deoxynulceotidyl transferase nick‐end‐labeling (TUNEL) staining in testes (n = 5). Scale bars, 20 µm

**Figure 7:**
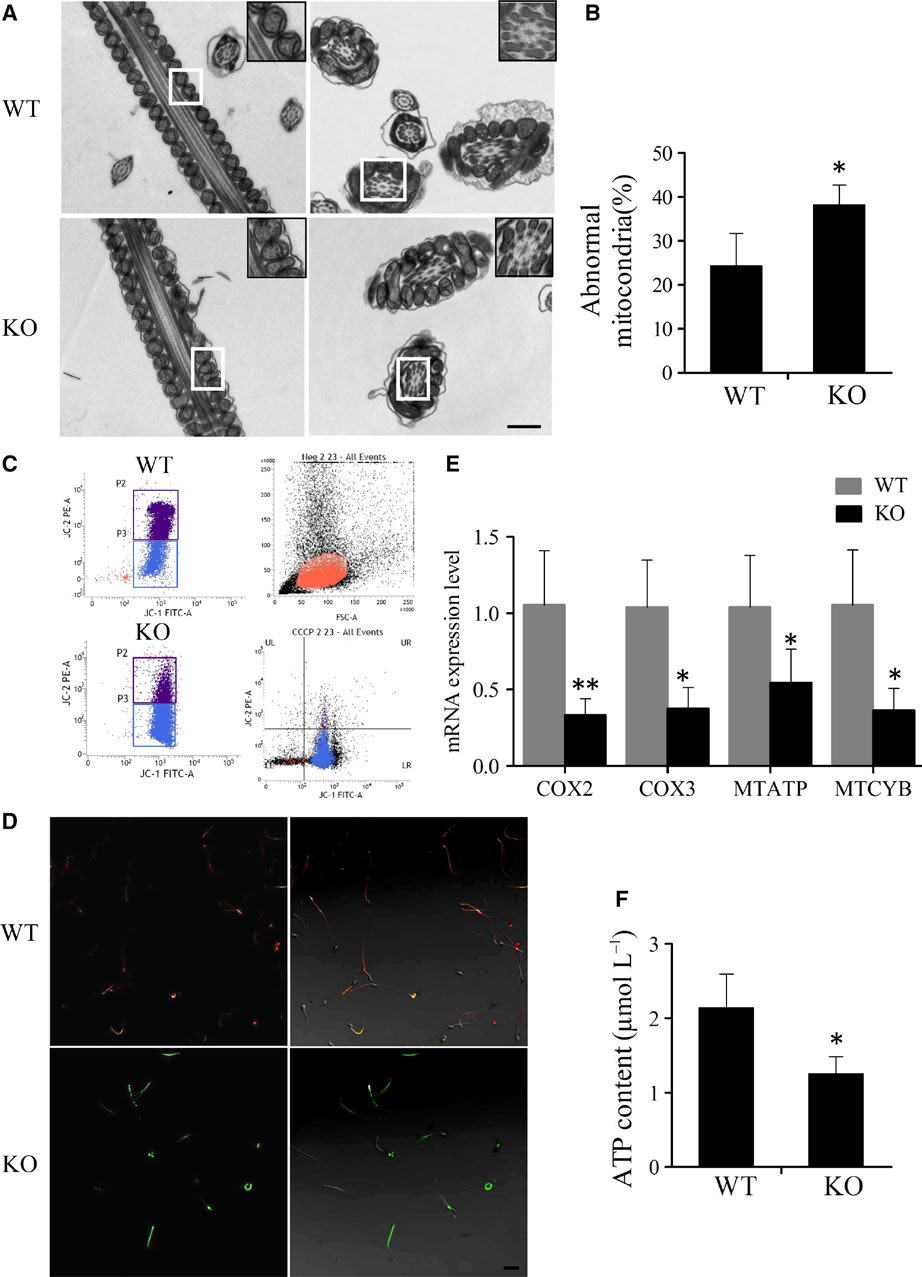
Assessment of ATP synthesis in sperm of wild‐type (WT) and knockout (KO) mice. (A) Transmission electron microscopy (TEM) images of sperm ultrastructures are shown. Scale bars, 0.5 µm. (B) Percentage of abnormal mitochondria in WT and KO male mouse sperm (n = 3). *P < 0.05. (C) The sperm samples from WT and KO mice were treated with the lipophilic cationic dye JC‐1 and were checked for their corresponding mitochondrial membrane potential (MMP) through flow cytometry analysis. P2, red‐stain sperm; P3, green‐stain sperm. Neg and carbonyl cyanide 3‐chlorophenylhydrazone were respectively used as a negative control and positive control respectively. (D) Fluorescence microscope observation of sperm after JC‐1 staining to determine the difference in MMP between WT and KO group. The red fluorescence sperm were normal and the green sperm had a low MMP. Scale bars, 50 µm. (E) Detection of mRNA expression levels of key markers in the mitochondrial electron transfer chain between WT and KO male mice sperm (n = 5). *P < 0.05, **P < 0.01. (F) Measured levels of ATP between WT and KO male mice sperm (n = 5)

**Figure 8:**
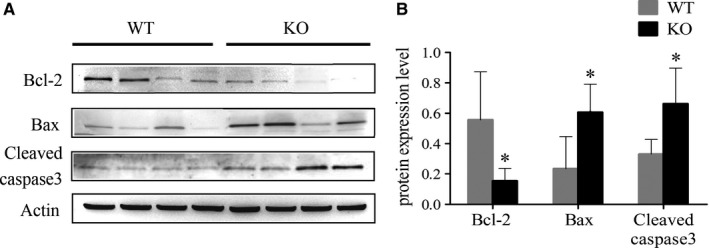
Assessment of apoptosis in sperm of wild‐type (WT) and knockout (KO) mice. (A) Detection of apoptosis in sperm samples from WT and KO mice using western blotting (n = 4). (B) Gray intensity analysis showing the expression level of pro‐ and anti‐apoptotic markers. *P < 0.05.

**Figure 9:**
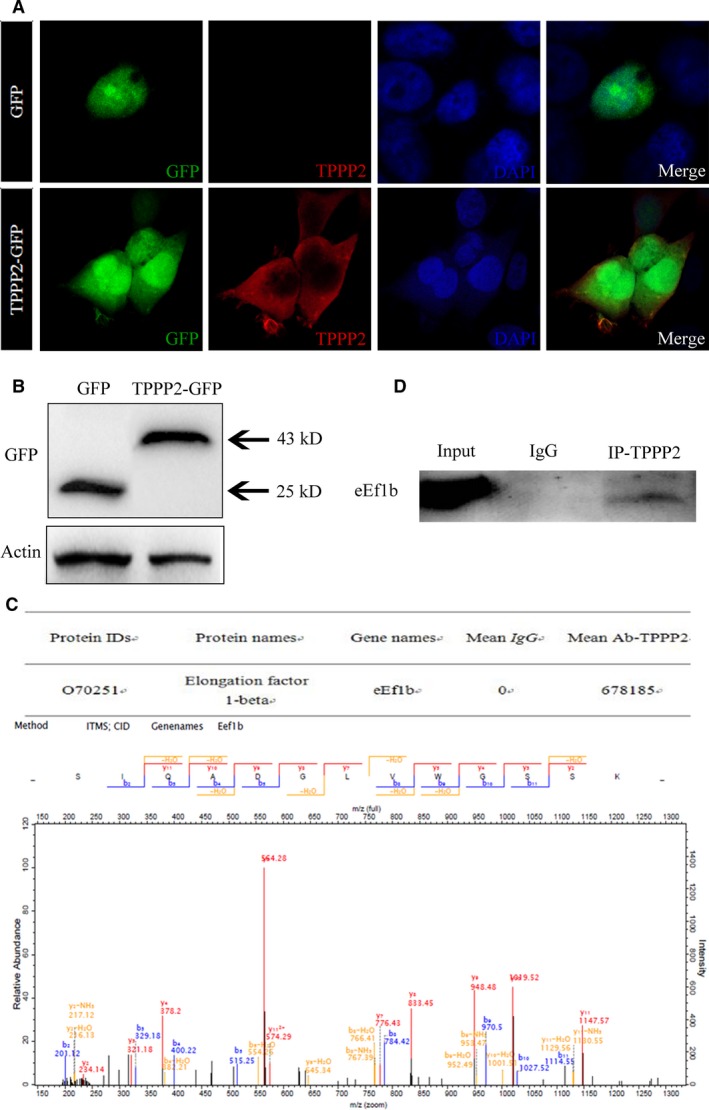
The discovery of TPPP2 interaction protein. (A) Localization of green fluorescent protein (GFP) and TPPP2‐GFP in HEK293T cells. Cells were stained with rhodamine phalloidin and observed using fluorescence microscopy 48 h after transfection. (B) The level of TPPP2 in HEK293T cells was verified with western blotting. (C) Immunoprecipitation of TPPP2‐GFP coimmunoprecipitated eEF1b, as assessed using mass spectrometry. (D) Immunoprecipitation of TPPP2 coimmunoprecipitated eEF1b in testes

To determine that the TPPP2 protein was not expressed in the testis and sperm of *Tppp2* ^−/−^ male mice, a series of western blotting, immunohistochemical and immunofluorescence assays were conducted. The results showed that the *Tppp2* ^−/−^ mouse testis and sperm had no TPPP2 protein expression (Figures 4, 5, 6, 7, 8, 9C‐F).

### 3.4 TPPP2 deficiency impaired sperm motility and count, thus affecting fertility

Mating tests followed by continuous monitoring for six months revealed that *Tppp2* ^−/−^ males exhibited a subfertile capacity (Figure 5A) (*P* < 0.001). Each month's fertility status was analysed and the status of fertility reduction in *Tppp2* ^−/−^ males was found to be persistent (Figure 5B) (for the first month: *P* < 0.05; for the second, fifth and sixth month: *P* < 0.01; for the third and fourth month: *P* < 0.001). CASA analysis revealed that the sperm counts and sperm motility of *Tppp2* ^−/−^ male mice were significantly lower than those of the controls (Figure 5C‐E) (for the progressive motility and sperm counts: *P < *0.05; for the total motility: *P < *0.01).

The weight and size of the testis did not differ significantly between the WT and *Tppp2* ^−/−^ mice (Figure 6A‐B). They both had intact spermatogenic tubules and spermatogenic cells at all stages could be seen, which were normal in shape and arranged in an orderly manner without shedding (Figure 6C). The number of spermatocytes and round spermatids were counted and there was no significant difference between WT and *Tppp2* ^−/−^ mouse testes (Figure 6D). In addition, apoptosis in the *Tppp2* ^−/−^ mouse testes was not significantly increased compared with that in the WT controls (Figure 6E).

### 3.5 Abnormal mitochondria and impaired ATP synthesis in *Tppp2* ^−/−^ male mice sperm

To seek the reason for the decreased sperm motility in the *Tppp2* ^−/−^ male mice, we first compared the ultrastructure of the sperm from the *Tppp2* ^−/−^ and wild‐type groups using transmission electron microscopy. The results revealed that the *Tppp2* ^−/−^ mice sperm tails contained normal outer dense fibres and a highly ordered structure, termed the “9 + 2” axoneme, which appeared no different to those of WT mice sperm. However, the mitochondria of *Tppp2* ^−/−^‐ mice sperm were irregular, with a significantly increased proportion of sperm lacking inner mitochondrial membrane (IMM) cristae when compared with those of the wild‐type mice (Figure 7A‐B) (*P < *0.05).

JC‐1 dye has been used to monitor MMP. High membrane potential was associated with emission at 590 nm (red) and low membrane potential at 530 nm (green). Detection of MMP showed that MMP in *Tppp2* ^−/−^ mice sperm was lower than that in wild‐type mice sperm (Figure 7C‐D).

The IMM contains the complexes for the electron transfer chain (ETC), which orchestrates the oxidative phosphorylation process and is associated with the ATP generation. We thus detected the mRNA expression levels of four key markers of the ETC complexes, that is, cytochrome C oxidase (COX)2, COX3, mitochondrial cytochrome B (MTCYB) and mitochondrial ATP synthase (MTATP). Real‐time PCR showed that the mRNA levels of all these four markers in *Tppp2* ^−/−^ mice sperm were significantly reduced when compared with those in the control mice (Figure 7E) (for the mRNA levels of MTATP and MTCYB: *P < *0.05; for the mRNA levels of COX2 and COX3: *P < *0.01).

The above results suggested that ATP synthesis might be disturbed in the sperm of *Tppp2* ^−/−^ mice. We detected the ATP content in the sperm of *Tppp2* ^−/−^ and WT mice, the results indeed showed significantly decreased ATP content in *Tppp2* ^−/−^ group (Figure 7F) (*P < *0.05). Thus, the significantly decreased sperm motility in *Tppp2* ^−/−^ mice sperm was probably caused by impaired ATP synthesis.

### 3.6 Sperm apoptosis was increased in *Tppp2* ^−/−^ male mice

A decline of MMP is regarded as a sign of early cell apoptosis. It is therefore necessary to examine the apoptosis state of sperm in *Tppp2* ^−/−^ mice. We focused on the intrinsic (mitochondria‐dependent) pathway, which involves both pro‐ and anti‐apoptotic members of the Bcl‐2 family. The results showed that the expression levels of Bax and cleaved caspase 3 in *Tppp2* ^−/−^ mice sperm were significantly increased, whereas the expression level of Bcl‐2 was significantly decreased (Figure 8) (*P < *0.05).

### 3.7 The identification of TPPP2 interacting proteins

A plasmid overexpressing TPPP2 with a GFP label was transfected into HEK293T cell lines. After transfection of the TPPP2 expression plasmid, the cellular proteins were extracted and anti‐GFP antibodies were used to detect the transfection efficiency using western blotting. The results showed that TPPP2 protein was diffusely expressed in the cytoplasm of HEK293T cells (Figure 9A‐B). An immunoprecipitation experiment was carried out with proteins extracted from HEK293T cells transfected with the TPPP2‐GFP plasmid and the samples obtained by precipitation were identified using mass spectrometry, which identified eEf1b (eukaryotic translation elongation factor 1 beta) (Figure 9C). The interaction between TPPP2 and eEf1b was verified in testes using immunoprecipitation and western blotting (Figure 9D).

## 4 DISCUSSION

The study of spermatogenesis/sperm function regulation is a research hotspot in the field of reproductive medicine. An orderly and specifically expressed gene/protein network may play an important role and a series of genes have been identified as related to spermatogenesis/sperm function.^15^, ^16^, ^17^, ^18^ In this study, we paid particular attention to a tubulin polymerization‐promoting protein family member, TPPP2, which is likely to play an important role in male reproduction.

Our results indicated that TPPP2 was specifically expressed in male reproductive organs, including testes and epididymis. In testes, TPPP2 was restrictively expressed at stages IV‐VIII of the seminiferous epithelial cycle, corresponding to the spermatids at steps 15‐16 of spermiogenesis. At this time the morphology of elongated spermatids has basically formed. In the epididymis, TPPP2 was expressed in sperm and was localized in the middle piece of the sperm tail. Such a specific expression pattern might indicate an important role of TPPP2 in sperm function.

To determine whether TPPP2 plays a role in sperm function, we conducted preliminary validation in sperm in vitro using anti‐TPPP2 antibody blocking experiments. The results revealed that after TPPP2 was inhibited, human sperm motility was significantly decreased, with a significantly lower ATP content in sperm, whereas the capacitation and acrosome reaction were not affected. Similar results were observed in mice and this adverse effect appears to further affect the in vitro fertilization rate. Therefore, the preliminary in vitro results suggested that TPPP2 could play an important role in sperm function by affecting energy production, leading to the observed changes in sperm motility. There is no doubt that the motility of sperm is directly related to fertilization and only a certain amount of normal progressively motile sperm can cross the barriers in the female genital tract and ensure fertilization of the egg. As mentioned above, male factors account for about 50% of the infertility causes. The prevalence of these male infertility cases was 18.71% for asthenozoospermia and 63.13% for asthenozoospermia associated with oligo‐ and/or terato‐zoospermia.^4^ However, the exact pathogenesis of oligoasthenozoospermia is not clear. TPPP2 is likely to be a potential pathogenic factor in male infertility. To explore the roles of TPPP2 in sperm function, we conducted further studies using *Tppp2* ^−/−^ mice.

Using *Tppp2* ^−/−^ mice, we demonstrate that TPPP2 is crucial for male fertility as the litter size of *Tppp2* ^−/−^ male mice declined significantly. Through further analysis, a significant decrease in sperm motility with a significantly lower ATP content was observed in *Tppp2* ^−/−^ mice, which was consistent with the results in vitro. In addition, we found that lack of TPPP2 in males caused a significantly reduced sperm count.

Sperm motility is an important parameter associated with fertility. Sperm motility is dependent on its specific structures (such as microtubules, outer dense fibres and mitochondria) and normal energy production. Ultrastructure analyses revealed that the microtubules and outer dense fibres in the *Tppp2* ^−/−^ mice were normal. However, impaired mitochondria structure in *Tppp2* ^−/−^ mice was observed with a significantly increased proportion of mitochondria lacking cristae compared with those of the control. The mitochondria are wrapped around the outer circumference of the middle piece of the sperm tail, forming the mitochondrial sheath structure. Mitochondria are considered important organelles for energy production in sperm. Therefore, mitochondrial abnormalities in *Tppp2* ^−/−^ male mice are very likely to be the underlying cause for its decreased sperm motility, suggesting that TPPP2 might play an important role in maintenance of mitochondrial structure and function of sperm, which is also consistent with its location in the middle piece of the sperm tail.

Mitochondria are characterized by four defined interconnected compartments: the outer mitochondrial membrane, the inner mitochondrial membrane (IMM), the intermembrane space and the mitochondrial matrix. The IMM is usually convoluted presenting several cristae and the number, structure and extension of IMM cristae may have functional consequences. Located in the IMM, the complexes of the ETC orchestrate the oxidative phosphorylation process. Electron transport through the ETC complexes generates a transmembrane electrochemical gradient that drives ATP synthase to produce ATP. The MMP is the main component of the transmembrane electrochemical gradient and thus changes associated with the MMP are critically important in studies regarding mitochondrial function.^32^ On this basis, we presume that the lack of IMM cristae in *Tppp2* ^−/−^ male mice has a significant influence on the quality and function of sperm. To confirm this conjecture, we conducted a series of tests.

After staining with JC‐1,^33^ a significantly decreased MMP in *Tppp2* ^−/−^ male mice sperm was found, suggesting that mitochondrial function was impaired. In addition, we detected several key markers of ETC complexes including COX2, COX3, MTCYB and MTATP, which are all correlated with ATP synthesis.^34^, ^35^, ^36^ Their significantly decreased expression levels in *Tppp2* ^−/−^ male mice sperm suggested that abnormal mitochondria severely affected the energy production and motor function of spermatozoa.

As for the lower sperm counts in *Tppp2* ^−/−^ male mice, we also explored the possible reasons. We first eliminated the possibility of spermatogenesis disorder in testes by counting spermatogenic cells. The number of spermatocytes and round spermatids were similar between *Tppp2* ^−/−^ and wild‐type male mice. Besides, our results also revealed no increase in TUNEL‐positive cells observed in the testis of *Tppp2* ^−/−^ male mice. As the decline of MMP is also an iconic event of early apoptosis,^37^ it is therefore necessary to verify whether the sperm are undergoing apoptosis. We focused on the intrinsic (mitochondria‐dependent) pathway, which involves, for example, both pro‐ and anti‐apoptotic members of the Bcl‐2 family and especially on the general apoptotic features, as there is clearly much more information at that level.^32^ The Bcl‐2 family is the main regulator of the mitochondrial apoptosis pathway activating a series of downstream genes to regulate apoptosis.^38^, ^39^ Bcl‐2 is an anti‐apoptotic protein whereas Bax promotes apoptosis and is the main mediator of mitochondrial pathways.^40^ A series of regulations of the mitochondrial apoptosis pathway eventually leads to the activation of caspase 3, which induces apoptosis.^41^ Our results showed that the expression levels of Bax and cleaved caspase 3 increased, whereas the expression level of Bcl‐2 decreased in *Tppp2* ^−/−^ mice sperm, which showed that the deletion of TPPP2 might activate the mitochondrial apoptotic pathways leading to lower sperm counts in *Tppp2* ^−/−^ male mice.

In summary, TPPP2 deficiency impaired male fertility, which may relate to the decrease of ATP synthesis and the increase of sperm apoptosis. In contrast to TPPP1 and TPPP3, which are linked to microtubules, TPPP2 distributes homogeneously within the cytosol of cells. This indicates that instead of affecting microtubules, TPPP2 functions in another way. Here, considering that TPPP2 has no domains with known function, we identified its potential interaction protein, eEf1b, to assist in explaining the mechanism of action of TPPP2. EEf1b, a subtype of eEF1, plays an important role in the extension of protein translation.^42^ We speculate that by affecting the function of eEf1b, TPPP2 might intervene in the translation of a series of related proteins, which participate in or regulate, the structure and function of mitochondria, thus affecting ATP production and apoptosis of spermatozoa.

This study used a *Tppp2* KO mouse model and human sperm samples to demonstrate the important role of TPPP2 in male fertility. Our results demonstrated that TPPP2 is essential for sperm function. TPPP2 is likely to be a potential pathogenic factor in male oligoasthenozoospermia. The detailed mechanism of TPPP2’s involvement in regulating sperm function will be clarified in further studies.

## CONFLICT OF INTEREST

The authors confirm that there are no conflicts of interest.
