# Supplementary material for: Deficiency of TPPP2, a factor linked to oligoasthenozoospermia, causes subfertility in male mice
Source: J Cell Mol Med. 2019 Jan 24;23(4):2583–94. doi: 10.1111/jcmm.14149 (PMC6433727; doi:10.1111/jcmm.14149)
Supplement: Supplementary file 1 [file JCMM-23-2583-s001.doc]

**Supplemental Table 1. Primer sequences and target fragment size of each gene**

| Gene name | Primer sequences (5’-3’) | Size (bp) |
| --- | --- | --- |
| TPPP2 | CTCCCCCAGCAATTGACCAT  TCAAAGCGCTCCTTGTGAGT | 382 |
| COX2 | AATTGCTCTCCCCTCTCTACG  GGTGCCCTATGGTTTTAACG | 83 |
| COX3 | AGGCCACCACACTCCTATTG  AGGCCACCACACTCCTATTG | 143 |
| MTCYB | ATTCCTTCATGTCGGACGAG  ATTCCTTCATGTCGGACGAG | 98 |
| MTATP | CTCACTTGCCCACTTCCTTC  GTAAGCCGGACTGCTAATGC | 114 |
| β-actin | TGACCCAGATCATGTTTGAGACC  ATAGATGGGCACAGTGTGGG | 142 |
